# Lack of an Association between Passive Smoking and Incidence of Female Breast Cancer in Non-Smokers: Evidence from 10 Prospective Cohort Studies

**DOI:** 10.1371/journal.pone.0077029

**Published:** 2013-10-18

**Authors:** Yuan Yang, Fan Zhang, Laura Skrip, Yang Wang, Shengchun Liu

**Affiliations:** 1 Department of Cardiovascular Medicine, the First Affiliated Hospital of Chongqing Medical University, Chongqing, China; 2 School of Public Health and Health Management, Chongqing Medical University, Chongqing, China; 3 Department of Epidemiology of Microbial Diseases, Yale School of Public Health, New Haven, Connecticut, United States of America; 4 Department of Endocrine and Breast Surgery, the First Affiliated Hospital of Chongqing Medical University, Chongqing, China; National Taiwan University, Taiwan

## Abstract

**Background:**

Several case-control studies have suggested that passive smoking may increase the incidence of female breast cancer. However, the results of cohort studies have been inconsistent in establishing an association. The present study evaluated the association between passive smoking and incidence of female breast cancer through a meta-analysis of prospective cohort studies.

**Methods:**

Relevant articles published before August 2012 were identified by searching the electronic databases PubMed, Embase, and Web of Science. Pooled relative risks (RRs) were determined with either a fixed or random effects model and were used to assess the strength of the association. Sensitivity and subgroup analyses according to ethnicity, menopausal status, and the period and place of exposure to passive smoking were also performed.

**Results:**

Ten prospective cohort studies involving 782 534 female non-smokers were included in the meta-analysis and 14 831 breast cancer cases were detected. Compared with the women without exposure to passive smoking, the overall combined RR of breast cancer was 1.01 (95% confidence interval: 0.96 to 1.06, P = 0.73) among women with exposure to passive smoking. Similar results were achieved through the subgroup analyses. No evidence of publication bias was observed.

**Conclusion:**

The results suggest that passive smoking may not be associated with increased incidence of breast cancer. However, the present conclusion should be considered carefully and confirmed with further studies.

## Introduction

Breast cancer contributes significantly to physiological and psychological comorbidities among women worldwide. In 2011, an estimated 230,480 new cases of invasive breast cancer affected women in the US and breast cancer is expected to account for 29% (226,870) of all new cancer cases in 2012 in this group [1], [2]. Among the environmental or lifestyle factors involved in the etiology of breast cancer, tobacco smoking may be one of the leading preventable risk factors [3].

In the past two decades, the health risks associated with cigarette smoking and exposure to tobacco smoking have been widely investigated by both basic science researchers and epidemiologists. Tobacco smoke contains polycyclic aromatic hydrocarbons, aromatic amines, and N-nitrosamines [4], all of which potentially lead to breast cell carcinogensis. However, the antiestrogenic effect of smoking has also been associated with reduced risk of breast cancer [5]. The antiestrogenic effect of smoking has likewise been associated with both an increased risk of osteoporosis and early onset of natural menopause among female smokers [6], [7]. Compared with women who had never smoked, Luo and colleagues [8] found that the incidence of breast cancer was increased by 9% among former smokers and by 16% among current smokers; what’s the worse, the risk of breast cancer was increased by 35% among the active smokers who smoked at least 50 years.

Numerous observational studies [8]–[17] have been conducted in an attempt to find the association between passive smoking and incidence of breast cancer. Passive smoking, or exposure to environmental tobacco smoke (ETS), has been associated with significant physiological harm related to that experienced by active smokers [18]–[19]. Yet, the results from studies specifically considering the association between passive smoking and breast cancer incidence have been inconsistent and suggested positive [12], [15], [16], inverse [10], and null associations [9], [11], [13], [14], [16], [17]. This observation was the initial motivation of the current study. In addition, there were two reasons to conduct a meta-analysis that only included prospective cohort studies. First, case-control studies tend to be influenced by more confounding and bias than studies with a prospective cohort design, and a meta-analysis combining both study designs could be affected by these issues. Second, a previous meta-analysis [14] combined several cohort studies and reported that passive smoking had no significant effect on breast cancer risk. However, compared with women who never exposed to smoking, the results combined with retrospective studies (most of them were case-control studies) [14] showed that the incidence of breast cancer was increased by 21% (95%CI: 11%–32%, P<0.05) among women with exposure to smoking, which was different with the results of prospective studies. In that analysis, evidence was limited because one of the eight included studies [20] only reported on breast cancer mortality; one study was case-control in design [21]; and another two studies [22], [23] that were included have since been updated with extended follow-ups and larger samples [15], [17]. Therefore, with the recent accumulation of additional evidence [8], [11], [13], [16], our goal was to evaluate the association between passive smoking and incidence of breast cancer by conducting a meta-analysis of prospective cohort studies.

## Materials and Methods

### Search strategy

The current study was conducted according to the PRISMA (Preferred Reporting Items for Systematic Reviews and Meta-Analyses) Statement [24]. Relevant studies were identified through PubMed, Embase, and Web of Science databases by using the following search terms: “*passive smoking*” or “*secondhand smoke*” or “*tobacco smoke pollution*” or “*environmental tobacco smoke*” and “*breast cancer*” or “*breast neoplasm*” or “*breast carcinoma*.” We also sought additional studies by reviewing the reference lists of included articles, reviews, conference abstracts, and the bibliographies of expert advisors. All articles identified through this search strategy had been published on or before August 27, 2012.

### Selection criteria

Titles and abstracts of all relevant papers were reviewed. The studies were chosen if they met all of the following criteria: (i) the study was designed as a prospective cohort study; (ii) the exposure was restricted to passive smoking; (iii) the articles provided relative risk (RR) estimates and the corresponding 95% confidence intervals, the size of the baseline samples, number of cases, follow-up years, confounders for adjustment, or other information that can help to infer the results; (iv) when multiple publications reported on the same or overlapping data, the study that was most recent or based on the largest population was selected; (v) the publication language was limited to English and Chinese. Reviews, meeting abstracts, editorials, and commentaries were excluded from our analysis.

### Data extraction

Data were extracted independently by two reviewers (Zhang F. and Yang Y.). Consensus was reached by discussion. A third party (Liu S.) was involved when necessary. The following information was extracted from each article: first author, year of publication, study location, ethnicity of subjects, study period, duration of follow-up, source population, mean age of the baseline sample, number of cases, assessment of passive smoking, ascertainment of cases, adjustment for covariates, relative risk (RR) and the corresponding 95% confidence interval.

### Assessment of risk of bias

Two authors (Zhang F, Yang Y) independently assessed the quality of each study based on the Newcastle-Ottawa-Scale (NOS). The NOS was developed for quality assessment of non-randomized observational studies, including both case-control studies and cohort studies [25]. A “star system” was developed under the NOS and was applied in the present analysis to judge each included study on the following: selection of the study groups, between-group comparability, and choice of outcome. Using the scale, we assigned a number of stars to each study. A study was awarded a maximum of one star for each numbered item within the Selection and Outcome categories. A maximum of two stars were assigned for Comparability. The total NOS star count ranged from zero to nine. Decisions were compared and consensus was reached, with a third party (Liu S) participating in quality assessment when necessary.

### Statistical analyses

Relative risk (RR) was used to measure the association between passive smoking and incidence of breast cancer. Two studies [13], [17] reported stratified risk estimates by place of exposure to passive smoking; one study [10] reported stratified risk estimates by number of household smokers; and one study [8] reported stratified risk estimates by husband's smoking status. We combined these estimates using the method reported by Hamling [26] and then used the pooled estimates for the overall meta-analysis. In the study by Lin et al [13], the control groups in the subgroup analyses of exposures to smoking at home and in public places were different, according to this, we treated this two subgroup analyses as two studies.

Heterogeneity of RR across studies was assessed using the Cochran's χ^2^-based Q test and the I-squared test. Heterogeneity was not considered as significant when *P*>0.10 or I2<50%. If no significant heterogeneity was found, the pooled RR estimate of each study was calculated using the fixed effects (Mantel-Haenszel) model [27]. Otherwise, the random effects (DerSimonian and Laird) model was used. Stratification analyses by ethnicity, menstrual status and place of passive smoking were conducted to reduce the heterogeneity and get more accurate results. We also performed additional meta-analyses that included only those studies with subjects experiencing passive smoking in childhood or adulthood, and then only those studies with follow-up of more than 10 years. The sensitivity analysis was conducted by excluding (i) the studies followed up less than five years, (ii) the studies with less than 100 cases, and (iii) the studies with NOS score less than 7. It was also conducted by using a leave-one-out approach to take into account variations in the assessed study quality.

Egger's linear regression test [28] and the Begg's rank correlation test [29] were used to assess potential publication bias. All statistical tests were conducted using STATA software (Version 11). A P-value of 0.05 for any test or model was considered to be statistically significant, except where otherwise specified.

## Results

### Literature search

**Figure 1:**
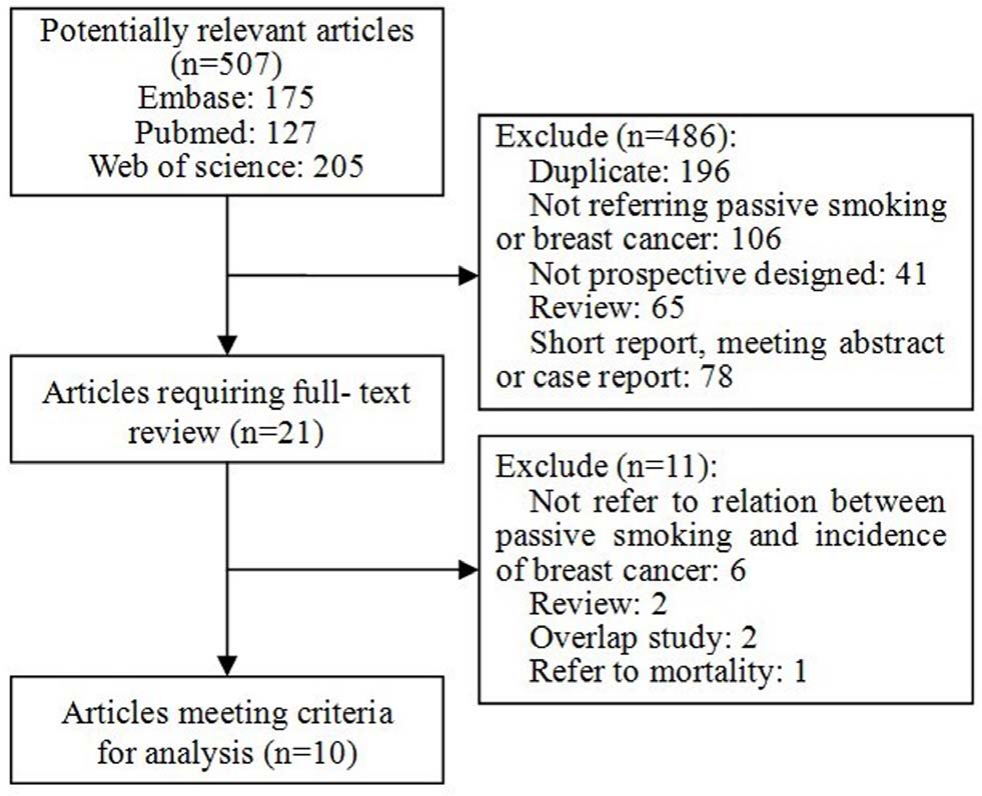
Flow chart of literatures selection. Flow chart shows literature search for prospective cohort studies of passive smoking in relation to incidence of breast cancer.

We initially retrieved 507 citations from PubMed, Web of Science and Embase databases. After title and abstract screening, most of the publications were excluded, mainly because they were duplicate records, reviews, cross-sectional or case-control studies, or not relevant to our study. Among the 21 studies included for full-text review, six [20], [30]–[35] were excluded from the analysis because they did not refer to the association between passive smoking and incidence of breast cancer. Two more articles [22], [23] were excluded since we were using articles that provided more recent data on the same studies but with extended follow-up [15], [17]. An additional two reviews [36], [37] and one study [20] referring to breast cancer mortality were excluded. A total of 10 studies were ultimately included in the analyses and all of them were published in English language. (**Figure 1**)

### Eligible studies

All of the included 10 studies were published between 1999 and 2011. Three studies were conducted in the United States, three in Japan, one in the United Kingdom, one in Korea, and two were multi-country studies. Six studies enrolled Caucasian participants and four studies' participants were of Asian background. At the study baseline, a total of 782 534 female non-smokers were involved. 14 831 cases were detected during the follow-up period. The length of follow-up ranged from 3.5 to 24 years (mean 10.2 years). The ascertainment of passive smoking varied across studies, with the majority basing exposure on self-report or interviewer-administered questionnaires. Most of the studies confirmed disease status according to ICD-9, ICD-O-2, medical records or pathology reports. The time of exposure to smoking were reported mainly by childhood [8], [13], [15], [16], adulthood [8], [13], [15], or the lifetime [9]–[12], [14], [17]. However, the kind of exposure included at passive smoking in the home [8]–[11], [13], [14], [17], a public place [13], or the workplace [8], [15], [17]. Several studies [12], [14], [15] were concerned with the premenopausal women, and some [8], [12], [14], [15] on postmenopausal women, and the rest included studies were not analyzed separately according to menstrual status. (**Table 1**)

**Table 1:** The characteristics of the included prospective cohort studies.

Studies	Country	Period	Ethnicity	Duration (years)	Population^#^	Number of all cases	Age (years)	NOS score	Menstrual status	Exposure		Adjustment for Covariates
										Time	Place	
1999, Jee	Korea	1994–1997	Asian	3.5	160,130	138	40–88	8	NA	NA	Home	Age, husband's age, husband's occupation, husband's vegetable consumption, residency, socioeconomic status
2001,Nishino	Japan	1984–1992	Asian	9.0	9,675	67	>40 y	7	NA	NA	Home	Age
2005, Gram	Norway, Sweden	1991–1992	Caucasian	9.3	102,098	1130	30–50	7	NA	NA	Home	Age, age at menarche, age at first birth, alcohol, BMI, family history of breast cancer, hormonal contraceptive use, menopausal status, number of children
2005,Hanaoka	Japan	1990–1999	Asian	10.0	20,191	162	40–59 y	8	Premenopausal or postmenopausal	NA	NA	Age, employment status, education level, BMI, family history of breast cancer, history of past benign breast disease, age at menarche, number of births, menopausal status, hormone use and alcohol consumption.
2008,Lin	Japan	1988–1990	Asian	12.0	32,023	196	40–79 y	8	NA	Childhood or adulthood	Home or public place	Age, area, BMI, family history of breast cancer, alcohol, daily walking, age at menarche, age at the first birth, menopausal status, number of births and use of sex hormones.
2008,Pirie	UK	1996–2001	Caucasian	3.5	224,917	2,344	50–64 y	7	Premenopausal or postmenopausal	Childhood or adulthood	NA	Age, BMI, age at first birth, age at menarche, region of residence, socio-economic group, physical activity, alcohol consumption, menopausal status, parity, hormone use.
2009,Reynolds	US	1997	Caucasian	10.0	57,523	1,754	NA	6	Premenopausal or postmenopausal	Childhood or adulthood	Home or workplace	Age, race, family history of breast cancer, age at menarche, pregnancy history, lifetime duration of breastfeeding, physical activity, alcohol consumption, BMI, menopausal status with use of hormone therapy.
2011,Chuang	Multi-countries*	1992–1998	Caucasian	10.0	98,938	3,411	25–70 y	8	NA	Childhood	NA	Age, sex, and study center, and adjusted for education, baseline alcohol drinking, BMI, physical activity, vegetable intake, fruit intake, non-alcoholic energy intake, and adulthood passive smoking, age at menarche, parity, ever use of oral contraceptives, and menopausal status
2011,Luo	US	1993–1998	Caucasian	10.3	41,022	3,520	50–79 y	8	Postmenopausal	Childhood or adulthood	Home or workplace	Age, race, parity, education, BMI, physical activity, alcohol intake, family history of breast cancer, hormone use
2011,Xue	US	1982–2006	Caucasian	24.0	36,017	2,109	30–55 y	7	NA	NA	Home or workplace	Age, family history of breast cancer, history of benign breast disease, BMI, BMI at age 18 years, height, alcohol consumption, age at menarche, parity, age at first birth, physical activity, oral contraceptive use, menopausal status, hormone use, and age at menopause.

BMI: Body mass index; CI: Confidence interval; NA: Not available; NOS: Newcastle-Ottawa-Scale; RR: Relative risk; US: United States.

* Multi-countries contain Sweden, Denmark, Norway, the Netherlands, UK, France, Germany, Spain, Italy, and Greece.

# Number of non-smoking women.

All 10 studies included adjustment for more than three variables, such as age, ethnicity, body mass index (BMI), menstrual status, family history of breast cancer, hormone use, socioeconomic status, alcohol, etc. The details were shown in the **Table 1**. After assessment of risk of bias using the NOS, all studies received from six to eight stars which means the quality of the studies were moderate to high.

### Quantitative synthesis

**Figure 2:**
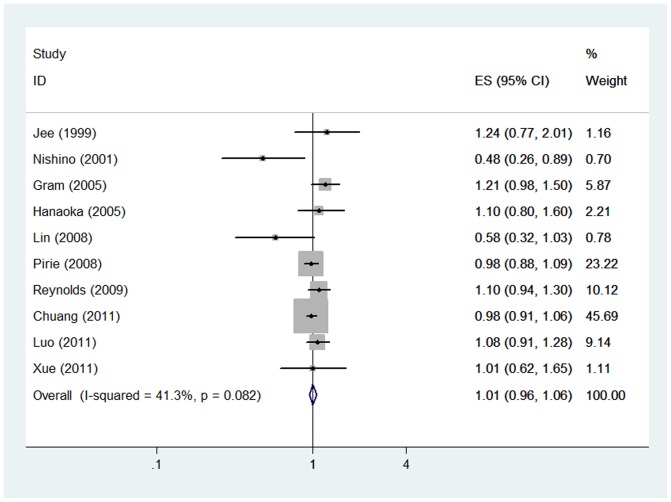
Forest plot of overall pooled RR. Forest plot shows association between passive smoking and incidence of breast cancer. CI: confidence interval; RR: relative risk.

Based on the combined results of the 10 cohort studies, among women who had never smoked, compared with those who had never been exposed to passive smoking, women exposed to passive smoking were not significantly associated with increased incidence of breast cancer (RR = 1.01, 95%CI: [0.96, 1.06], P = 0.73) under the fixed effect model (Heterogeneity: I^2^ = 41.3%). (**Figure 2**)

A subgroup analysis was conducted according to ethnicity and no significant associations were found in either Asian populations (RR = 0.82, 95%CI: [0.54, 1.27], P = 0.38) or Caucasian populations (RR = 1.02, 95%CI: [0.96, 1.07], P = 0.58). Subgroup analysis according to menopausal status further revealed that passive smoking was not associated with the incidence of breast cancer (Premenopausal: RR = 1.11, 95%CI: [0.55, 2.24], P = 0.78; Postmenopausal: RR = 1.01, 95%CI: [0.85, 1.20], P = 0.90). Whether the period of exposure occurred in childhood or adulthood did not affect the findings of a null association between passive smoking and risk of breast cancer (Childhood: RR = 1.09, 95%CI: [0.99, 1.20], P = 0.10; Adulthood: RR = 1.03, 95%CI: [0.91, 1.17], P = 0.63). No association was observed upon combining the results of studies grouped according to whether exposure occurred at home or in the outside (Home: RR = 0.96, 95%CI: [0.81, 1.14], P = 0.67; Workplace or public place: RR = 1.01, 95%CI: [0.93, 1.10], P = 0.76). Furthermore, when we combined the studies with follow-up of at least 10 years, no statistically significant relationship was observed between passive smoking and the incidence of breast cancer (RR = 1.01, 95%CI: [0.95, 1.07], P = 0.80). (**Table 2**)

**Table 2:** Subgroup analyses of meta-analysis.

Groups	No. of studies	RR	95% CI	P values	I^2^	Analysis models
Asian	4	0.82	[0.54, 1.23]	0.38	67.3%	Random effects
Caucasian	6	1.02	[0.96, 1.07]	0.58	5.6%	Fixed effects
Premenopausal	3	1.11	[0.55, 2.24]	0.78	82.4%	Random effects
Postmenopausal	4	1.01	[0.85, 1.20]	0.90	64.6%	Random effects
Childhood	4	1.09	[0.99, 1.20]	0.10	0.0%	Fixed effects
Adulthood	2	1.03	[0.91, 1.17]	0.63	0.0%	Fixed effects
Home	7	0.96	[0.81, 1.14]	0.67	55.5%	Random effects
Workplace	4	1.01	[0.93, 1.10]	0.76	0.0%	Fixed effects
Follow-up≥10 y	6	1.01	[0.95, 1.07]	0.80	15.9%	Fixed effects

CI: Confidence interval; RR: Relative risk.

### Sensitivity analysis

Sensitivity analyses were conducted to examine the influence of various exclusion criteria on the overall risk estimate and to explore potential sources of heterogeneity in the association between passive smoking and risk of breast cancer. Exclusion of the two studies that had a follow-up time of less than five years yielded a pooled RR of 1.02 (95%CI: [0.91, 1.15], P = 0.71, I^2^ = 51.5%). Restricting the analysis to studies that had more than 100 breast cancer cases (only the study by Nishino et al was excluded) yielded a pooled RR of 1.02 (95%CI: [0.96, 1.07], P = 0.58), without evidence of between-study heterogeneity (I^2^ = 17.5%; P = 0.29). The pooled RR was 1.00 (95%CI: [0.95, 1.05], P = 0.99, I^2^ = 43.4%) when the study conducted by Reynolds et al [14] was excluded, which got lowest NOS score. Furthermore, exclusion of any single study did not materially alter the overall combined RR, which then ranged from 1.00 (95% CI: [0.95, 1.05], P = 0.94) to 1.04 (95% CI: [0.97, 1.11], P = 0.34).

We also conducted the analysis by combining those six studies in which, passive smoking was more comprehensively assessed and defined specifically as exposure to smoking by parents, partners, or coworkers [8], [13]–[17]. However, the result was not materially changed with pooled RR of 1.00 (95% CI: [0.95, 1.05], P = 0.96, I^2^ = 14.5%).

### Publication bias

**Figure 3:**
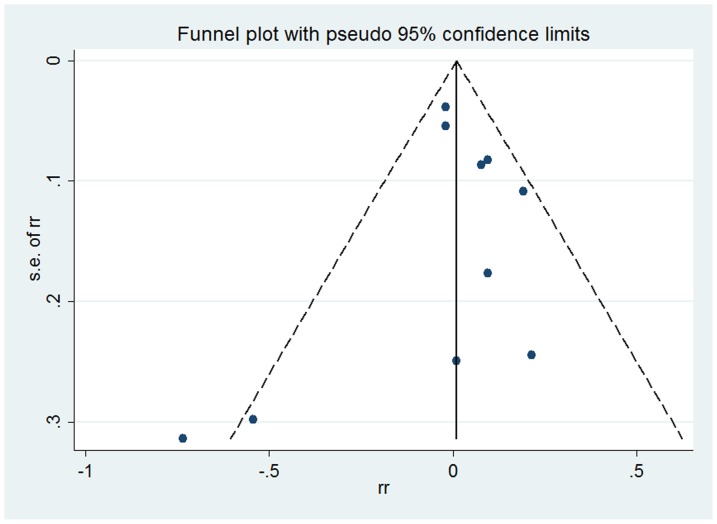
Funnel plot of included studies. Funnel plot shows association between passive smoking and incidence of breast cancer.

Funnel plot was generated to assess the possibility of publication bias. As shown in **Figure 3**, the shape of funnel plot was relatively symmetrical thus suggesting there was no significant publication bias. Begg's rank correlation test and Egger's linear regression test also supported no obvious publication bias (*P*_Begg_ = 0.37, *P*_Egger_ = 0.79).

## Discussion

There is rapidly growing interest in the public health implications of passive smoking. Our study focused on the risk of breast cancer in female non-smokers with passive exposure. This meta-analysis found no association between passive smoking and incidence of female breast cancer. This finding was consistent in all analyses, including subgroup analyses separately investigating the association in Asian versus Caucasian populations, premenopausal versus postmenopausal women, individuals with childhood versus adulthood exposure, and individuals with household versus workplace exposure. This null association was also observed in our meta-analysis considering the six studies with extended follow-up of 10 years or longer.

Previous original studies investigating this association have generated a variety of results. For instance, the California Environmental Protection Agency (CalEPA) [38] reported that exposure to tobacco smoke at younger ages, specifically in the premenopausal years, was associated with a 68% increased risk of breast cancer, compared to women without exposure to passive smoking. However, most of the studies included in CalEPA research were retrospective in design. Recently, Luo and colleagues [8] suggested that postmenopausal women with the most extensive exposure to passive smoking (10 or more years of exposure in childhood, 20 or more years of exposure as an adult at home, and/or 10 or more years exposure as an adult at work) experienced a 32% increase in the risk of breast cancer compared to postmenopausal women without exposure. The California Teachers Study (CTS) [15] and the Nurses' Health Study [17] both failed to find a positive association between passive smoking and incidence of breast cancer. Due to limitations in the data available from original studies, our study could not include a dose-response analysis by passive smoking exposure metrics.

Several possible reasons may explain the lack of statistically significant findings in the present review. First, underlying toxicological and biological mechanisms of tobacco smoke are likely responsible for any association between passive smoking and increased incidence of breast cancer. A study by Petrakis et al detected carcinogens in breast fluid obtained through standard nipple aspiration techniques among nonlactating women [39] and thus provided evidence for a relationship between passive smoking and increased incidence of breast cancer. Other studies, however, have found that oestriol excretion was significantly lower in smokers than in non-smokers [5], [40], which indicated that smoking has an antiestrogenic effect. Since use of estrogen replacement therapy has been shown to result in a significantly higher risk of breast cancer [41], [42] and higher estrogen levels have been associated with increased incidence of breast cancer, the antiestrogenic effect of smoking could be treated as a rational possible explanation for the results of our meta-analysis.

Another possible explanation for the observed null association was that all of the included cohort studies did not consider genetic confounders. Several breast cancer susceptibility genes, such as BRAC1, BRAC2 [43] and CHEK2 [44], [45], have been detected in recent decades. Additionally, Conlon et al [46] suggested that the effect of smoking on breast cancer may be differentially modified by the N-acetyltransferase 2 (NAT2) phenotype. Among NAT2 fast acetylators, subjects with a history of more than 20 pack-years were nearly two times as likely (OR: 1.93, 95%CI: 1.01–3.69) to develop breast cancer as compared to individuals with a history of less than 20 pack-years; however, no association was detected among NAT2 slow acetylators. Another recent meta-analysis [47] further suggested that NAT2 polymorphisms contribute to the risk of breast cancer when smoking history is taken into account. NAT1 genetic polymorphisms were also considered to be sensitive to smoking history in the etiology of breast cancer. A previous study [48] revealed that the relationship between smoking and risk of breast cancer was modified by the NAT1 or NAT2 genotype among postmenopausal women. Zhang et al [49] also found that the association between NAT1*10 allele and risk of breast cancer was mainly limited to former smokers (OR = 3.30, 95% CI: 1.20–9.50). Several conventional confounders—such as age, age at menarche, age at first birth, parity, family history of breast cancer, body mass index (BMI), oral contraceptive use, menopausal status, hormone use, and age at menopause—were included for analysis in our study, which were adjusted in most of the included studies for eliminating the potential bias. However, genetic distributions, especially for genes with expression affected by tobacco smoke should be considered in the future research.

Subgroup analysis showed that passive smoking could not increase/decrease the incidence of breast cancer in premenopausal women, which was different from the findings of Hanaoka et al [12] and Pirie et al [14]. Hanaoka and colleagues [12] found that passive smoking increased breast cancer risk (RR = 2.6; 95%CI: [1.3, 5.2]) in premenopausal women. However, in the Million Women Study [14], inverse association was shown in premenopausal women (RR = 0.54, 95%CI: [0.30, 0.99]). No significant association was found in the study conducted by Reynolds et al [15]. Maybe this was the potential explanation why the heterogeneity was high in this subgroup. Combining those results, the association between passive smoking and incidence of breast cancer was not statistically significant (RR = 1.11, 95%CI: [0.55, 2.24], P = 0.78). The possible explanation was that the genetic factor may impact the effect of tobacco smoking on breast cancer [47]–[49]. In the subgroup analysis of postmenopausal women, although Hanaoka et al [12] found that passive smoking may decrease the incidence of breast cancer (RR = 0.6, 95%CI: [0.4, 1.0]), this study did not support this point by combining 4 studies (RR = 1.01, 95%CI: [0.85, 1.20], P = 0.90). So, future studies should consider other potential impact factors, not only the menstrual status.

Substantial heterogeneity was observed among the included studies, which was not surprising given the differences in study population characteristics, assessment of passive smoking, duration of follow-up, and adjustment for confounding factors. Our sensitivity analyses suggest that studies with fewer than 100 cases probably contributed to the observed heterogeneity. The study [10] with the smallest numbers of both participants and cases increased the possibility that chance accounted for their results; the RR reported in this study was evidently lower than that reported in others, thus indicating that passive smoking could decrease the risk of breast cancer. Exclusion of any single study in the sensitivity analysis revealed that the overall combined RR ranged from 1.00 to 1.02 which indirectly indicated that the result of our study was robust.

A major strength of our study is that all the included original studies used a prospective cohort design, which minimizes selection bias. Moreover, the lack of an association between passive smoking and risk of breast cancer did not change with sensitivity analyses based on rigorous exclusion criteria and various subgroup analyses. Additionally, by pooling evidence from multiple studies and generating a large overall sample size, we have enhanced statistical power to provide more precise and reliable risk estimates than provided by individual studies.

In this meta-analysis, several limitations may have influenced our findings. One potential limitation involved variations in the how passive smoking was measured in the included studies. Most studies assessed passive smoking through self-completed questionnaires containing various items. Three [9]–[11] of them assessed active smoking among the non-smokers' family members—husband, parents, or other cohabitating partners—and they defined a passive smoker as someone living with an active smoker. One study [12] focused on exposure to passive smoking not only at home, but also at the workplace. In the other six studies [8], [13]–[17], passive smoking was measured more accurately, and the combined result of those studies was not substantially changed (RR = 1.00, 95% CI: [0.94, 1.05], P = 0.89, I^2^ = 6.3%). A second limitation is the substantial heterogeneity among studies. However, through the sensitivity analysis, we were able to identify the major source of heterogeneity and demonstrate through a leave-one-out procedure that our findings are robust despite it. Thirdly, a dose-response analysis could not be conducted due to limitations in the data of original studies. Addressing the level of exposure may result in different findings. For instance, Luo and colleagues [8] found that the postmenopausal women with most extensive exposure to passive smoking had a 32% increased risk of breast cancer compared with those who had never been exposed to passive smoking. In an attempt to consider length of exposure, we combined the studies with 10 or more years of follow up in an additional, separate analysis; however, we found no significant association between passive smoking and incidence of breast cancer in this analysis. Finally, language bias was introduced since our search included studies written in English or Chinese. According to the above limitations, our conclusion should be considered carefully and confirmed with further studies that concentrate on questionnaire design and a dose-response analysis of passive smoking.

## Conclusions

The meta-analysis of prospective cohort studies suggests that passive smoking may not be associated with increased incidence of breast cancer. However, the present conclusion should be considered carefully and confirmed with further studies.

## Supporting Information

Checklist S1 **PRISMA Checklist.**
(DOC)
